# Decoding Virulence and Revisiting the Evolution of Klebsiella pneumoniae

**DOI:** 10.7759/cureus.109253

**Published:** 2026-05-20

**Authors:** Janahvi A Pimpalkhare, Satyajeet Pawar, Satish Patil

**Affiliations:** 1 Department of Microbiology, Krishna Institute of Medical Science, Krishna Vishwa Vidyapeeth (Deemed to be University), Karad, IND

**Keywords:** antimicrobial resistance, carbapenem resistance, classical klebsiella pneumoniae, global dissemination, hvkp, hypermucoviscosity, hypervirulent klebsiella pneumoniae, siderophores, virulence factors

## Abstract

A unique and clinically relevant pathotype known as hypervirulent *Klebsiella pneumoniae* (hvKp) is distinguished by its increased virulence and capacity to cause serious community-acquired infections in otherwise healthy people. HvKp was first identified in the Asia-Pacific region and is now progressively disseminating worldwide, including India and other regions. This study provides a summary of the historical evolution, microbiological characteristics, phenotypic and genotypic determinants, global dissemination, and developing antimicrobial resistance patterns linked to hvKp. The convergence of hypervirulence and multidrug resistance, including carbapenem-resistant hvKp strains, complicates therapeutic management and poses a significant threat to public health. Strengthened surveillance, molecular diagnostics, and antimicrobial stewardship are essential to mitigate the expanding impact of hvKp globally.

## Introduction and background

The genus Klebsiella, belonging to the family Enterobacteriaceae, was established by Vittore Benedetto Antonio Trevisan in 1885 in honor of the German bacteriologist Theodor Albrecht Edwin Klebs (1834-1913), recognized for his contributions to establishing the microbial basis of infectious diseases. The first *Klebsiella pneumoniae *was discovered as a human pathogen in the late 19th century. In 1882, Carl Friedländer described this organism as an encapsulated bacillus following its isolation from the lungs of patients who had died of pneumonia. It was initially termed “Friedländer’s bacillus” and was later assigned to the genus Klebsiella in 1886 [1,2]. The taxonomy of the genus Klebsiella underwent substantial revision. Initially, it was divided into three species, *K. pneumoniae*, *K. ozaenae*, and *K. rhinoscleromatis*, primarily based on associated clinical syndromes [3]. Advances in numerical taxonomy and molecular characterization have led to multiple reclassifications, with current genomic studies recognizing *Klebsiella pneumoniae* as part of a species complex comprising closely related taxa such as *K. pneumoniae*, *including K. variicola* and *K. quasipneumoniae* [3]. By the early 1980s, *K. pneumoniae* had emerged as the most clinically significant species [4,5].

*Klebsiella pneumoniae* is a clinically important Gram-negative, encapsulated, facultative anaerobic bacillus from the order Enterobacterales [6,7]. Also, it is a well-recognized cause of a wide spectrum of nosocomial and community-acquired infections, including pneumonia, septicemia, and urinary tract infections, and is associated with multidrug-resistant (MDR) infections [6,7]. The multidrug-resistant (MDR) nature of *Klebsiella pneumoniae* is mainly due to the acquisition of resistance genes that act against multiple antibiotic classes. β-lactam resistance is driven by carbapenemase genes such as blaKPC, blaNDM, and blaOXA-48-like, which break down carbapenems, and extended-spectrum β-lactamases like blaSHV and blaTEM, which degrade penicillins and cephalosporins [8,9]. These genes are often carried on plasmids, allowing them to spread quickly between strains and across different regions [3,4]. This organism is widely distributed across diverse ecological niches, including soil, water, plant surfaces, and the mucosal surfaces of humans and animals, where it commonly exists as a commensal colonizer [8]. A defining structural characteristic of *K. pneumoniae* is its prominent polysaccharide capsule, which acts as a major virulence determinant by providing resistance to complement-mediated killing and phagocytosis [9]. A key feature underlying the evolutionary success of *Klebsiella pneumoniae* is its ability to acquire and integrate foreign genetic material, which has led to the emergence of two major pathotypes, classical *K. pneumoniae* (cKp) and hypervirulent *K. pneumoniae* (hvKp), through specific genomic changes, including mutations, allelic variations, and the acquisition of virulence-associated genes [10].

Classical *K. pneumoniae* is widely reported as a cause of hospital-acquired pneumonia and urinary tract infections, particularly in elderly or immunocompromised individuals. HvKp exhibits enhanced pathogenicity and has the capacity to cause severe community-acquired infections in healthy individuals as well. Increasingly, hvKp has been linked with healthcare-associated infections. Beyond its established role as a major cause of pyogenic liver abscess (PLA), hvKp is also linked to metastatic and multisite infections, including meningitis, epidural abscess, endophthalmitis, lung abscess, pneumonia, non-hepatic abscesses, septic arthritis, osteomyelitis, and necrotizing fasciitis [11,12]. Historically, community-acquired *K. pneumoniae *pneumonia was characterized by sudden onset, high fever, hemoptysis producing the classical “currant jelly” sputum, and distinctive radiological features such as cavitary lesions and bulging interlobar fissures. By the late 20th century, however, the incidence of classical community-acquired *K. pneumoniae* pneumonia declined in several developed countries, particularly in the United States [13,14].

**Figure 1:**
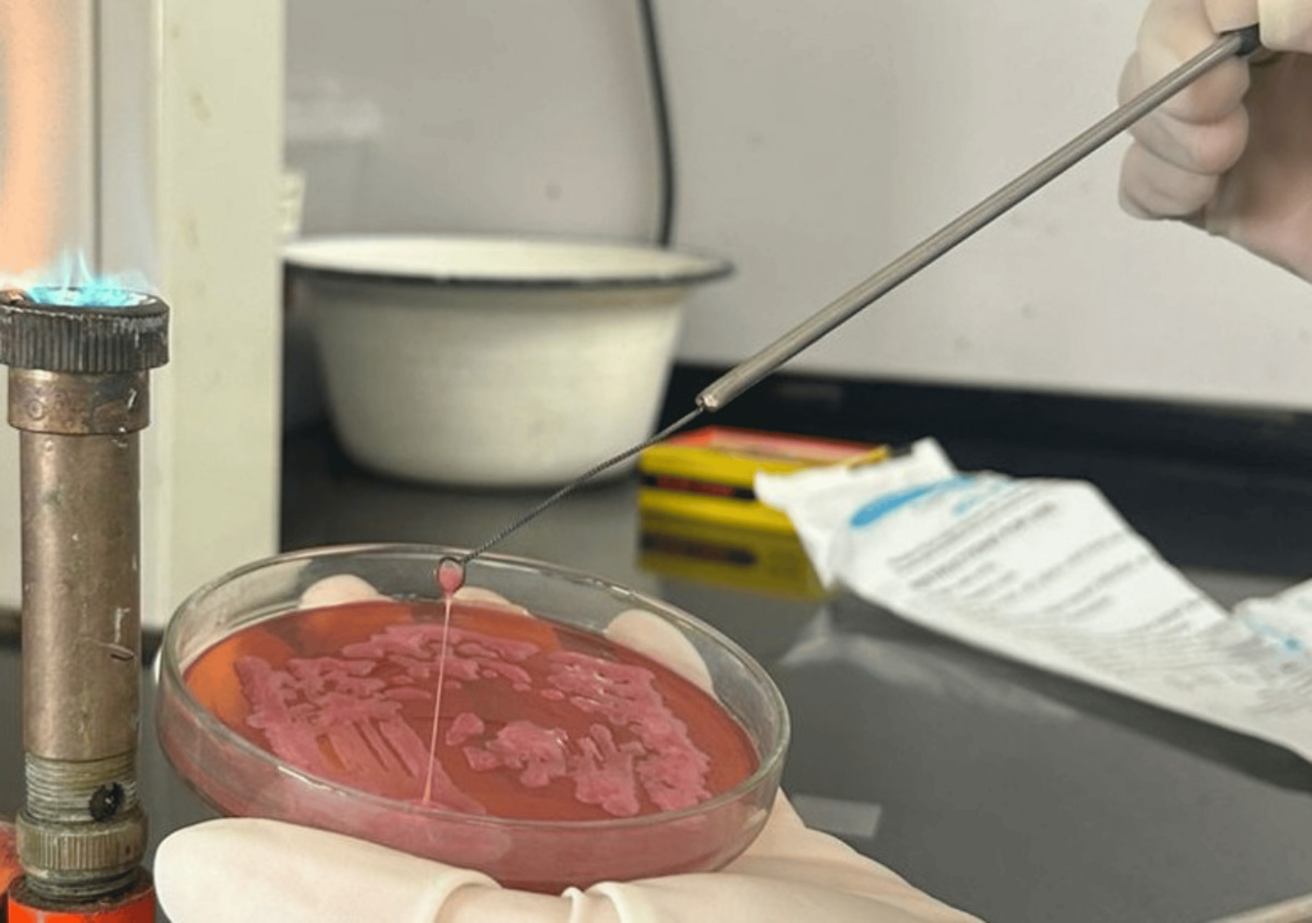
String test positive for HvKp. The image is captured by the author of this study. HvKp: hypervirulent *Klebsiella pneumoniae*

Hypervirulent* K. pneumoniae* (hvKp) exhibits a hypermucoviscous phenotype driven by increased capsule production, which can be identified using the string test (Figure 1). In this test, a colony is stretched with an inoculation loop, and the formation of a viscous strand longer than 5 mm indicates a positive result [15,16]. This characteristic is a key marker of virulence and is associated with factors encoded within the accessory genome, particularly genes involved in capsule regulation such as rmpA and magA, which are strongly linked to hvKp strains and play a significant role in its pathogenicity, especially in strains reported from Asian regions [10,17-22].

In addition to capsular hypermucoviscosity, structural components such as fimbriae further contribute to the virulence of *K. pneumoniae*. Two principal fimbriae have been described as follows: type 1 and type 3 fimbriae, encoded by the fim (including fimH) and mrkABCD gene clusters, respectively. These structures play an important role in bacterial adhesion and biofilm formation, contributing to colonization and persistence within the host. Type 1 fimbriae are primarily involved in adhesion to epithelial surfaces, whereas type 3 fimbriae are strongly associated with biofilm formation on abiotic surfaces such as medical devices. Type 3 fimbriae are considered important virulence factors in hypervirulent *K. pneumoniae* (hvKp); however, their precise contribution to hypervirulence remains to be fully elucidated [5,19]. At the molecular level, the hvKp virulence plasmid encodes peg344, a putative metabolite transporter associated with hypervirulent strains. Although its precise biological function is not fully understood, peg344 has been linked to enhanced bacterial fitness in host environments. Studies have shown that when *K. pneumoniae* is cultivated in human ascites, peg344 expression may increase RNA abundance, suggesting a role in the uptake or transport of host-derived growth factors. Due to its consistent presence in hvKp-associated virulence plasmids, peg344 is considered a reliable biomarker for the identification of hypervirulent strains [10,17]. Hypervirulent *Klebsiella pneumoniae* (hvKp) has shown increasing global distribution, with early reports predominantly from the Asia-Pacific region, where it was first recognized as a major cause of community-acquired invasive infections. Over time, its spread has expanded worldwide, with cases now reported across Europe, the Americas, and parts of India, including Western Maharashtra [10,20,23,24].

This review synthesizes the evolution of hypervirulent *Klebsiella pneumoniae* (hvKp), focusing on its epidemiological patterns, genotypic determinants, and global dissemination, to provide a framework for understanding virulence acquisition and emerging antimicrobial resistance.

## Review

Search strategy

To prepare this review article on hypervirulent *Klebsiella pneumoniae* (hvKp), a comprehensive literature search was conducted using databases including Google Scholar, PubMed, Scopus, and ScienceDirect. The search strategy incorporated relevant Medical Subject Headings (MeSH) terms and keywords such as “hypervirulent *Klebsiella pneumoniae,*” “hvKp,” “virulence factors of *K. pneumoniae,*” “hypermucoviscosity,” “capsular serotypes,” “molecular epidemiology of hvKp,” “antimicrobial resistance in hvKp,” “hypervirulent carbapenem-resistant* K. pneumoniae,*” “carbapenem-resistant hvKp,” and “global dissemination of hvKp.” The literature search included studies published from 1984 to 2026, with earlier studies primarily included to provide historical context and more recent publications used to capture current advances in hvKp research. Peer-reviewed English-language studies focusing on hypervirulent *K. pneumoniae* and including relevant microbiological, molecular, epidemiological, or clinical data were included. Studies were excluded if they were non-peer-reviewed, lacked clear molecular or phenotypic characterization of hvKp, had insufficient data, or were not directly relevant to the objectives of this review.

The historical context

Hypervirulent Klebsiella pneumoniae

The recognition of hypervirulent *K. pneumoniae* (hvKp) arose from the identification of a distinctive clinical syndrome in Taiwan during the mid-1980s. In 1986, Liu et al. reported a notable case of community-acquired *K. pneumoniae* liver abscess associated with severe extrahepatic complications, including purulent meningitis, prostate abscess, and endophthalmitis, resulting in permanent visual loss despite intensive therapy [5].

This case was remarkable because it occurred in a previously healthy individual without conventional risk factors for classical Klebsiella infection, indicating a shift in the known disease pattern [5,10,25]. Throughout the 1980s and 1990s, increasing reports from the Asia-Pacific region described severe community-acquired infections caused by *Klebsiella pneumoniae*, consolidating recognition of this emerging hypervirulent entity [5,26]. The predominant clinical manifestation was pyogenic liver abscess, frequently accompanied by metastatic complications such as endophthalmitis, meningitis, and bacteremia [5,8,25]. Unlike traditional polymicrobial liver abscesses commonly attributed to *Escherichia coli*, this syndrome demonstrated a marked tendency for metastatic dissemination and affected healthy individuals [8,10].

Subsequent epidemiological studies confirmed that *Klebsiella pneumoniae* had become the leading cause of pyogenic liver abscess in Taiwan, establishing hvKp-associated pyogenic liver abscess (PLA) as an endemic condition in the region. Recognition of this distinct clinical phenotype prompted extensive investigation into the microbiological and genetic determinants of enhanced virulence, including accessory genomic elements that differentiate hypervirulent strains from classical *Klebsiella pneumoniae* [10].

Microbiological characteristics of hvKp

Numerous microbiological determinants contributing to the hypervirulent phenotype of *Klebsiella pneumoniae* have been thoroughly analyzed for more than four decades of its recognition. Principal factors linked to hvKp pathogenicity include capsular hyperproduction, the hypermucoviscous phenotype, siderophore-mediated iron acquisition systems, and additional virulence-associated components, such as lipopolysaccharide (LPS), colibactin, and fimbrial structures. A hallmark feature of hypervirulent*K. pneumoniae* isolates is their ability to cause severe invasive disease in otherwise healthy individuals [10].

Capsule Production

Capsular polysaccharide synthesis is a key virulence factor in *K. pneumoniae*, enabling evasion of host immune responses, including phagocytosis, complement-mediated killing, and antimicrobial peptides [4,5]. Serological classification of *Klebsiella pneumoniae* is based on capsule (K-antigen) typing, with at least 79 capsular types identified to date. Among these, eight serotypes are as follows: K1, K2, K5, K16, K20, K54, K57, and KN1. These are linked with hvKp, with K1 and K2 being the most commonly reported [10,17-22]. Capsule biosynthesis is controlled by genes placed within the chromosomal capsule polysaccharide synthesis (cps) locus, including wzi, wza, wzb, wzc, gnd, wca, cpsB, cpsG, and galF. Increased capsule production is further influenced by chromosomal regulators such as magA (also designated wzy-K1) and rmpA/rmpA2, which govern the mucoid phenotype. Additionally, plasmid-encoded virulence genes, including rmpA, rmpA2, and peg344, contribute to capsule hyperproduction and are variably present on virulence plasmids [10,17-22].

Siderophore Systems

Siderophores are low-molecular-weight iron-binding molecules that play a crucial role in bacterial iron acquisition, allowing survival in iron-limited host environments, a process known as nutritional immunity [10,17-22]. *K. pneumoniae* produces four main siderophores, enterobactin, yersiniabactin, aerobactin, and salmochelin, encoded by the ent, ybt, iuc, and iro gene clusters, respectively. Enterobactin, produced by most strains, has a strong affinity for iron but is often neutralized by the host protein, reducing its effectiveness during infection. Yersiniabactin contributes to bacterial survival under host-induced stress and supports virulence, although it is present in both classical and hypervirulent strains. Salmochelin, a glycosylated derivative of enterobactin, enhances iron acquisition by enabling evasion of host defense mechanisms. In contrast, aerobactin is strongly associated with hypervirulent *K. pneumoniae* (hvKp) and is frequently reported in these strains. It is considered a major virulence factor due to its efficient iron-scavenging ability and relative resistance to host defense mechanisms, particularly in systemic infections. The combined presence of aerobactin and salmochelin is a distinguishing feature of hvKp and highlights the importance of siderophore-mediated iron acquisition for its pathogenicity [10,17-22].

Lipopolysaccharide (LPS)

Lipopolysaccharide, a major structural component of the outer membrane of *K. pneumoniae*, comprises lipid A, an oligosaccharide core, and the O-antigen polysaccharide. These elements are encoded by the lpx, waa, and wb gene clusters, respectively. Lipopolysaccharide functions both as a defense against humoral immune defenses and as a potent activator of host inflammatory responses. Although LPS contributes to overall pathogenicity, its specific role in distinguishing hvKp from classical strains remains incompletely understood [10,21].

Antimicrobial resistance in hypervirulent *Klebsiella pneumoniae*

Hypervirulent *Klebsiella pneumoniae* was originally described as largely susceptible to commonly used antimicrobial agents, particularly in early Asia-Pacific reports of community-acquired invasive infections in otherwise healthy individuals [8,27]. Over the past decade, however, a marked epidemiological shift has occurred with the emergence of strains exhibiting both hypervirulence and multidrug resistance [10,28]. The following two related forms are recognized: hypervirulent carbapenem-resistant *K. pneumoniae* (hv-CRKp), in which hypervirulent strains acquire resistance, and carbapenem-resistant hypervirulent *K. pneumoniae* (CR-hvKp), in which resistant strains acquire virulence traits. Both represent the convergence of resistance and virulence, leading to highly severe and difficult-to-treat infections [20].

Genomic studies demonstrate that hvKp strains acquire resistance determinants primarily through plasmid-mediated horizontal gene transfer, particularly carbapenemase genes such as blaKPC, blaNDM, and blaOXA-48-like variants [5,28]. Of particular concern is the fusion of virulence plasmids, harboring loci such as iuc (aerobactin), iro, and regulators rmpA/rmpA2, with resistance plasmids, producing strains that are both highly invasive and extensively drug-resistant.

Reports from Asia, Europe, and the Americas confirm the increasing detection of these convergent strains in both healthcare-associated and community-onset infections [10,28]. Hypervirulent sequence type ST23 and other high-risk clones have now been identified carrying carbapenemase genes, indicating clonal expansion and global dissemination of resistant hypervirulent lineages [5,28]. In certain regions, hvKp isolates exhibit resistance not only to carbapenems but also to third-generation cephalosporins, fluoroquinolones, aminoglycosides, and occasionally polymyxins, substantially limiting therapeutic options [10,29].

Carbapenem-resistant hypervirulent *K. pneumoniae* (CR-hvKp)

The convergence of antimicrobial resistance with hypervirulence represents a major shift in *K. pneumoniae* epidemiology. Unlike classical multidrug-resistant strains that predominantly affect immunocompromised hosts, carbapenem-resistant hypervirulent *K. pneumoniae* (CR-hvKp) retains the ability to cause severe invasive infections in otherwise healthy individuals while exhibiting resistance to last-line agents [20,27,28]. Notably, these strains do not appear to exhibit the typical reduction in virulence associated with antimicrobial resistance, suggesting that resistance and pathogenicity can coexist without an apparent fitness disadvantage. This convergence has important clinical implications, as it is associated with more severe disease and limited treatment options, and represents a growing concern for both hospital and community settings [8,10].

Detection methods

The detection of hvKp cannot be determined only based on phenotypic techniques. When compared and concluded with genotypic approaches, they can produce the best findings.

Phenotypic Methods

The hypermucoviscous phenotype is assessed using the string test, as described above. In this method, a colony grown on solid media such as blood agar, nutrient agar, or MacConkey agar for 18-24 h at 35°-37°C is gently stretched using an inoculation loop. The formation of a viscous string of ≥5 mm is considered a positive result [21]. This method is regarded as a crude screening test, as results can vary depending on factors such as the type of media and incubation conditions. The interpretation is subjective, as some hypervirulent strains may not exhibit a positive result. Therefore, the string test should be used as a preliminary screening test and compared with molecular methods for accurate identification.

Tellurite resistance test: Tellurite resistance has been associated with hypervirulent *Klebsiella pneumoniae* (hvKp), as the virulence plasmid frequently carries tellurite resistance genes such as terW, which are linked to hypervirulence [21,30]. The tellurite resistance test is a simple screening method in which isolates are cultured on selective media supplemented with tellurite. In some studies, concentrations such as 0.1% potassium tellurite have been used to facilitate detection. On such media, tellurite-resistant strains produce characteristic black colonies, which may indicate the presence of hvKp [21,31,32].

Genotypic Methods

PCR: To identify hypervirulent *Klebsiella pneumoniae *(hvKp) strains, virulence biomarker genes such as rmpA and rmpA2, iucA, iucB, iroB, and peg344 can be amplified using PCR [16,23]. These virulence indicators provide molecular evidence to differentiate hvKp from classical *K. pneumoniae* (cKp) strains.

Multiplex PCR: Multiplex PCR involves generating multiple copies of a single or several target genes, followed by quantitative assessment of the amplified DNA using fluorescence emission. It has developed as a strong tool in microbiological diagnostics because of its high sensitivity, specificity, and speed of results [21].

Whole-genome sequencing: Detecting Kp, including pathotype and antibiotic-resistant genes, is crucial for the timely implementation of clinical therapies and infection control measures, especially for identifying multidrug-resistant (MDR) hvKp. Current detection methods have limits, necessitating the need for more efficient diagnostic instruments like next-generation sequencing (NGS) technology, which offers a high-output approach to microbial genomic investigation, processing millions of DNA fragments simultaneously to fully characterize pathogens and identify virulence factors and antibiotic-resistant genes. For this scope, NGS techniques such as Illumina (San Diego, CA: Illumina, Inc.) and Ion Torrent (South San Francisco, CA: Thermo Fisher Scientific) have been used to generate either short or long reads [21].

Distribution of hypervirulent *Klebsiella pneumoniae* (hvKp)

Western Maharashtra

Data regarding hvKp prevalence are limited in Western Maharashtra, but a recent study by Desai et al. in Pune, Maharashtra, indicates the presence of virulence-associated genes in clinical *Klebsiella pneumoniae* isolates from tertiary healthcare settings [24]. Although comprehensive epidemiological data specific to Western Maharashtra are lacking, identification of virulence markers in resistant isolates implies silent circulation and probable underrecognition, largely attributable to limited routine molecular surveillance.

Maharashtra and India

At both state and national levels, increasing recognition of hvKp has been reported across multiple regions of India. Whole-genome sequencing and hospital-based surveillance studies have identified diverse capsular serotypes and sequence types associated with hypervirulence [33]. Indian studies report variable prevalence depending on patient population and diagnostic criteria, with hvKp identified in both community-acquired and healthcare-associated infections [34,35].

The emergence of hvKp in India is increasingly supported by studies from tertiary care centers, including those conducted in Bhubaneswar (Odisha) and New Delhi, which document its presence in clinical isolates and its involvement in diverse hospital-based infections, underscoring its growing recognition within the Indian healthcare setting. Genomic studies from Vellore (Tamil Nadu) and Karnataka, along with findings from studies conducted across various regions of the country, show variation in virulence genes such as aerobactin and rmpA, suggesting that hypervirulent *Klebsiella pneumoniae* in India comprises multiple evolving lineages rather than a single dominant type [7,12,33,34]. These observations emphasize the need for coordinated molecular surveillance across Indian states to accurately define prevalence and transmission dynamics.

Asia-Pacific Region

Hypervirulent *K. pneumonia*e was first recognized in the Asia-Pacific region, particularly Taiwan and neighboring Pacific Rim countries, where community-acquired pyogenic liver abscess caused by hypervirulent strains became a defining clinical entity. Subsequent regional investigations established sustained endemicity in East and Southeast Asia. The Asia-Pacific region continues to report a higher prevalence of hvKp compared to many Western regions, although increasing detection elsewhere may partly reflect improved diagnostic awareness rather than true recent emergence [10,34,35].

Europe and the Americas

During the past decade, hvKp has been reported more frequently across Europe and North America. Initially regarded as sporadic or travel-associated, recent genomic studies suggest local transmission and establishment of hypervirulent lineages within healthcare environments. Particular concern is the emergence of carbapenem-resistant hypervirulent *Klebsiella pneumoniae *(CR-hvKp), which combines high invasiveness with restricted therapeutic options. Surveillance data suggest that convergence of virulence plasmids with carbapenemase genes is no longer geographically confined and has now been reported across multiple continents [10,20,25,36].

Global Perspective

**Figure 2:**
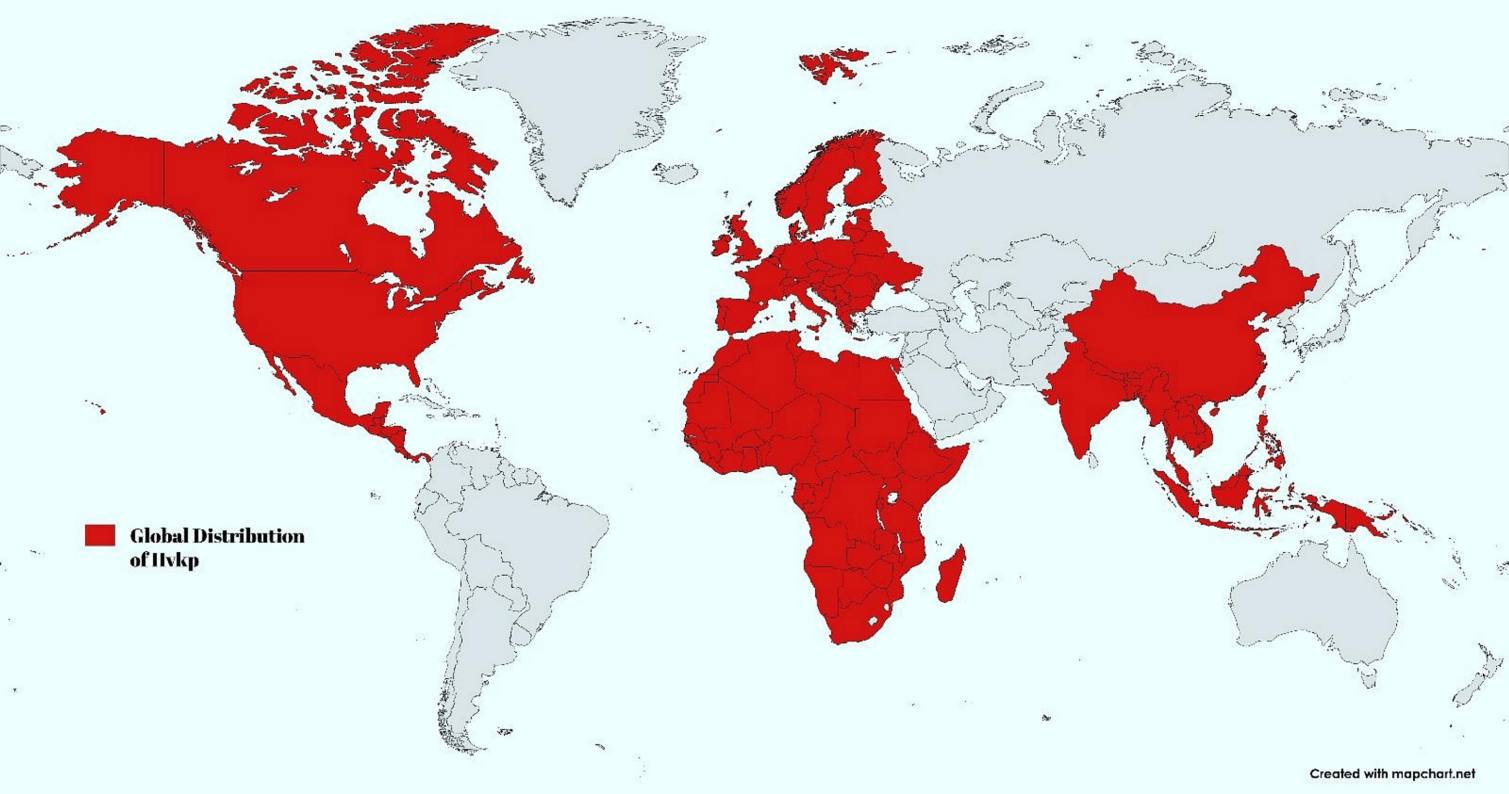
Spread of hypervirulent Klebsiella pneumoniae worldwide. The regions where hypervirulent *Klebsiella pneumoniae* has been documented in the literature are indicated by red color, suggesting that it is widely distributed throughout Asia, Europe, the Americas, and parts of Africa. This image is created by the author of this study using mapchart.net.

The World Health Organization has recognized hypervirulent *K. pneumoniae* as an emerging global health threat due to its expanding geographic distribution and increasing acquisition of antimicrobial resistance determinants. Reports now encompass Asia, Europe, the Americas, and parts of Africa, indicating widespread dissemination (Figure 2). This global spread is driven by multiple factors, including mobile genetic elements that facilitate horizontal gene transfer, international travel, and healthcare-associated transmission, along with the increasing convergence of virulence and resistance plasmids [35,37,38].

In addition, the emergence of carbapenem-resistant hypervirulent* K. pneumoniae* (CR-hvKp) has intensified concerns, as these strains combine high virulence with limited therapeutic options, contributing to increased morbidity and mortality [20,28,34]. The ability of these strains to spread across both community and healthcare settings further amplifies their public health impact [10,20,28,37].

Collectively, current evidence indicates that hvKp has transitioned from a regionally restricted clinical entity to a globally disseminated pathogen [5,10,20]. However, its true burden is likely underestimated, particularly in low- and middle-income countries, where limited access to molecular diagnostics and surveillance systems restricts accurate detection and reporting of hypervirulent strains [10,20].

Limitations

This review is based on previously published studies that vary in the molecular markers used to identify hypervirulent *Klebsiella pneumoniae *(hvKp), which may affect the consistency and comparability of findings. Limited region-specific epidemiological data, particularly from India and Western Maharashtra, restricts understanding of the burden and distribution of hypervirulent *Klebsiella pneumoniae*.

Recommendations

To enable early detection and precise identification of hypervirulent *Klebsiella pneumoniae* (hvKp), standardized laboratory diagnostic approaches integrating both phenotypic methods (such as string test and antimicrobial susceptibility testing) and genotypic techniques (including PCR-based detection of virulence genes such as rmpA, magA, and iuc) should be developed and implemented. Strengthening surveillance systems is essential to monitor hvKp prevalence, antimicrobial resistance trends, and transmission dynamics. This can be achieved through coordinated regional and global surveillance networks, incorporation of genomic epidemiology tools, and routine reporting of hvKp-associated infections.

Future directions 

Future research should focus on advancing rapid and accurate diagnostic technologies, particularly genome-based methods such as whole-genome sequencing, to facilitate early identification of hypervirulent strains. In addition, there is a need to develop targeted therapeutic strategies, including novel antimicrobial agents, combination therapies, and anti-virulence approaches, to effectively manage infections caused by emerging hypervirulent and multidrug-resistant strains. Further studies are also required to better understand the mechanisms underlying the convergence of virulence and antimicrobial resistance, which will be critical for informing clinical management and public health interventions.

## Conclusions

Hypervirulent *Klebsiella pneumoniae* has evolved from a geographically confined clinical entity into a globally disseminated pathogen of major clinical significance. Unlike classical strains, hvKp exhibits enhanced virulence, the ability to cause invasive and metastatic infections in healthy individuals, and a growing capacity to acquire antimicrobial resistance determinants. Emerging evidence from India, including Western Maharashtra, indicates increasing regional recognition and probable underestimation of its prevalence. The cooccurrence of hypervirulence with carbapenem resistance represents a critical turning point in *Klebsiella pneumoniae* epidemiology, combining high pathogenic potential with limited therapeutic options.

Genomic plasticity, plasmid-mediated acquisition of virulence and resistance determinants, and clonal expansion have facilitated its global dissemination. Despite increasing awareness, variability in diagnostic practices, and limited molecular testing likely obscure the true burden of hvKp. Coordinated genomic surveillance, early molecular detection of virulence markers, and strengthened antimicrobial stewardship programs are essential to contain its spread. As hvKp continues to evolve, proactive monitoring and integrated public health strategies will be crucial to prevent its progression into a more widespread and difficult-to-treat global threat.
